# Blood biomarkers for multiple sclerosis: neurofilament light chain and beyond

**DOI:** 10.1016/j.ebiom.2024.105214

**Published:** 2024-06-14

**Authors:** eBioMedicine

According to WHO, multiple sclerosis affects around 1.8 million people worldwide. It is an inflammatory demyelinating condition accompanied by neurodegeneration and characterised by an autoimmune attack on myelin, the lipid-rich material that protects nerves in the brain and spinal cord. Diagnosing multiple sclerosis is challenging, especially in early stages of the disease. Considering advances in therapeutic approaches for multiple sclerosis in the past three decades, biomarkers that can inform on prognosis and treatment efficacy are essential to adapt treatments to the individual characteristics of each patient.

Neurofilament light chain (NfL) assessment is becoming a useful tool to assist decision making in the management of neurodegenerative diseases, in combination with MRI. Neurofilaments are cytoskeletal proteins that are essential for the stability and radial growth of myelinated axons, ensuring optimal nerve-conduction velocity. Knowledge that neurofilaments are released into cerebrospinal fluid and blood upon axonal injury led to use of NfL, the smallest and most abundant neurofilament subunit, as a biomarker to assess brain damage. Assessment of concentrations of NfL can enable early detection of pathological processes, but also provide information about disease progression, prognosis, and treatment response, as discussed in a review published in *Nature Reviews Neurology*.

The concept that NfL could be useful as a biomarker in people with multiple sclerosis dates to 1998. In a study published in the *Journal of Neurology, Neurosurgery and Psychiatry*, Lycke and colleagues showed that, in 47 (78%) of 60 patients with relapsing–remitting multiple sclerosis, NfL was significantly increased in samples of cerebrospinal fluid. Moreover, there was a moderate association between NfL concentrations and Expanded Disability Status Scale score. Gisslén and colleagues subsequently showed, in a cross-sectional study published in *eBiomedicine* in 2016, that NfL concentrations in cerebrospinal fluid and in plasma were highly correlated. Although the study aimed to monitor brain damage in the context of HIV infection, it suggested that concentrations of NfL could be assessed in plasma without the need for lumbar puncture. To establish NfL as a convenient biomarker to assist with clinical decision making, reference ranges need to be defined. In a study published in *The Lancet Neurology* in 2022, Benkert and colleagues set up a reference database with age-adjusted and BMI-adjusted serum NfL concentrations based on samples from a general population of people from Europe and North America. However, as discussed in a Review in *eBioMedicine*, there is still work to be done to improve standardisation across testing platforms (eg, reference intervals, percentiles, and Z scores) and interpretation of results while considering potential confounders.

Although evidence indicates that serum NfL can be used to monitor treatment efficacy and identify individual patients at risk for a detrimental disease course, the full potential of NfL assessment is yet to be fully explored. For example, NfL can be combined with other biomarkers to improve risk stratification. In a study published in *eBioMedicine* in 2023, Hegen and colleagues investigated the additive predictive value for disease activity of serum NfL and κ-free light chain (κ-FLC), a biomarker associated with inflammation, in a cohort of 86 patients with a first demyelinating CNS event. Patients with increased κ-FLC index and serum NfL Z score had a 98% risk of relapse within 12 months, whereas patients in whom only one of these variables was increased had a risk of approximately 20–30% of relapse, suggesting that the combination of these two biomarkers improves identification of patients at high risk for early disease activity.

The value of NfL in people with non-relapsing progressive disease will also be interesting to assess. In a study published in *eBioMedicine* in 2023, concentrations of NfL were assessed in 1421 patients with relapsing multiple sclerosis and 596 patients with primary progressive multiple sclerosis. Results suggested that subtle NfL atypicalities could reflect underlying non-relapsing disease progression. Moreover, after suppression of relapse activity by ocrelizumab, persistently increased NfL, measured at week 24 or week 48 on treatment, was strongly associated with future disease progression in both groups of patients. Furthermore, the recognition of NfL as a suitable biomarker to assess treatment efficacy could accelerate the testing of new drugs, as highlighted by approval of tofersen by the US Food and Drug Administration in 2023 for amyotrophic lateral sclerosis associated with a mutation in the *SOD1* gene—partly on the basis of the drug’s ability to decrease NfL concentrations.

Although NfL is a promising blood biomarker in people with multiple sclerosis, there is still an important gap to be filled. As a non-specific biomarker, NfL has little diagnostic value, and increased NfL can be found in blood due to several neurological conditions. Hopefully, disease-specific blood-based biomarkers for the diagnosis of multiple sclerosis will soon be a reality. In a study published in *Nature Medicine*, Zamecnik and colleagues generated whole-proteome autoantibody profiles of 250 patients with multiple sclerosis and identified an autoantibody signature that was predictive of the disease in 10% of these patients. This autoantibody reactivity could be detected on samples collected at a mean of 5 years before symptom development and was shown to be highly specific for patients eventually diagnosed with multiple sclerosis in a separate incident cohort.

The management of multiple sclerosis has changed substantially in the past 30 years. The addition of cladribine, glatiramer, and rituximab to the WHO Essentials Medicine List in 2023 was an essential development to improve treatment access across the world. Although NfL cannot replace MRI, this relatively inexpensive biomarker could be an important advance to improve patient monitoring and help to reduce the global burden of the disease. At *eBioMedicine*, we welcome studies on biomarkers to assist on diagnosis, risk stratification, and therapy optimisation in patients with multiple sclerosis.

